# Commission of Lunacy on Two Sisters

**Published:** 1848-04-01

**Authors:** 


### COMMISSION OF LUNACY ON TWO SISTERS, MISS MARIA COLLINGS AND MISS AMELIA MARIA HORTENSIA COLLINGS,

On 20th December, 1847, at the Roebuck Tavern, Chiswick,

*Before* Francis Barlow, Esq., *Master in Lunacy.*

The jury was sworn. Mr. Temple appeared for Mr. and Mrs. Findlater, the petitioners.

Mr. Miller appeared for Maria Collings, (one of the alleged lunatics,) and also for Sir William Collings, Mrs. Manger Smith Collings, Mrs. Naftee, Mrs. Carrè, Mrs. Lukis, and Mrs. Guerin.

Mr. Temple (for the Commission).—You cannot so appear without the proper authority.

Commissioner.—You can only appear by the authority of the Court of Chancery, or with the authority of Miss Maria, the alleged lunatic. I cannot recognise you for any other party. If such were not the practice of the Court of Chancery, the estates of lunatics might soon be swallowed up.

Mr. Miller.—The other parties I appear for reside at Guernsey. Miss Maria was domiciled at Guernsey; she never lived elsewhere. The Cour Royale at Guernsey had control over her property. Her trustees reside at Guernsey. The Cour Royale had appointed an inquiry bearing the name of *Un Conseil de famille.* The property is vested in Mr. Joseph Collings, and the committee and guardians of the estate of this lady. Mr. Collings has placed her in the asylum, where she now is, and has exercised the proper control over her; and in order to be properly prepared, I am instructed to ask your Honour for an adjournment.

Commissioner.—She was placed in the asylum under the usual certificate.

Mr. Temple.—Yes, and it is only desired to adjourn, in order to change the proceedings.

COMMISSIONER.—The commission has been issued under the Great Seal, and I must therefore assume that all has been regular, and consequently, we cannot go into the question of regularity, as all appears *primâ facie* regular. You, Mr. Miller, say you appear for certain members of the family whom I cannot recognise. You say a commission has been issued in Guernsey, which is out of the jurisdiction of the Lord Chancellor. The court of Guernsey has no jurisdiction here. It is quite clear, when the commission was issued, that the party was within the jurisdiction of the Court of Chancery; though if the party were abroad, and had property in this country, it would be equally regular. The members of the family have no authority to appear before me. The Chancellor is now ill, but I cannot believe any application to him would have the effect of causing this commission to be set aside on the ground you urge. The utmost the Chancellor could do, would be to allow you to appear before me. You admit before me that she was of unsound mind, so as to require protection in Guernsey, and, therefore, that she required protection here. In point of law, we are not met to decide the question as to the issue of the commission in Guernsey. We have evidence that she requires protection here.

Mr. TEMPLE.—I appear for the only sister as the sole next of kin, who of course for form, was joined with her husband, Mr. Findlater, as petitioners. When they arrived here from Montpellier, in the south of France, where they had been residing, they found their sister in a lunatic asylum.

COMMISSIONER.—I think it a case for the jury.

Mr. MILLER.—I have an application to make on behalf of the lady alone. She wishes to be allowed an opportunity, I do not care how short, to bring forward evidence to meet this case. Her notice had been short; it had only been served upon her last Wednesday, the 14th of December, which makes it but four days. All the friends of this lady, excepting her sister and husband, reside in Guernsey, from whence she may have to bring witnesses. For any purpose of that kind, the notice is no doubt too short.

COMMISSIONER.—The parties are not bound to serve a notice at all.

Mr. MILLER.—Either a notice is necessary, or serving one is a perfect farce. No opportunity of seeing this lady has been afforded to me as her professional adviser.

COMMISSIONER.—Counsel does not necessarily see her. I think the lady has had sufficient notice; nearly a week is ample time for her to have appeared if she thought proper. I am not, therefore, disposed to adjourn at this moment, as we are assembled here at considerable expense to the estate, and I shall propose to go on, with perfect liberty to the learned counsel to apply for an adjournment at a later period of the day.

Mr. MILLER.—I cannot cross-examine the witnesses. I have no means, no materials.

COMMISSIONER.—The lady is in an asylum, by the act of the trustees, your clients.

Mr. TEMPLE.—Yes, she is in an asylum, from which she would, if sane, have been discharged from confinement by the visiting Commissioners. The application for the adjournment appears to be made by those who placed her there.

Mr. MILLER.—I only apply as a measure of justice, in a very important case.

COMMISSIONER.—At present we do not know what the case is. I will adjourn it at any period you show me it is a proper case for adjournment. As to cross-examining the witnesses, you shall have them back at any time.

Mr. MILLER.—I have no facts to examine upon.

Mr. TEMPLE.—If in the course of the inquiry you show the least necessity for an adjournment, I will press for the adjournment rather than object to it.

COMMISSIONER.—If after seeing the alleged lunatic I find it necessary that it should stand over, I will not of course object.

Mr. MILLER.—I am content to allow her to remain where she is for a few days to meet this case.

COMMISSIONER.—Under restraint, through parties for whom you say you appear; they are parties who put her there.

Mr. MILLER.—We might have gone to the Lord Chancellor in order to have it adjourned.

COMMISSIONER.—I suppose it is very unimportant as to what period it is carried back—should it be three or five years. You cannot show that she is in her right mind now. If you wish it, I will go and see her. I should do so if you were not here.

Mr. Miller.—I will put in the note sent by Miss Collings objecting to this case proceeding in haste.

Commissioner.—Is she here?

Mr. Moore (*Solicitor*).—She is not. She refuses to come. She objects to leave the asylum.

Mr. Miller.—I object to this matter proceeding in such haste.

Commissioner.—How do you appear? (*To Mr. Moore.*)

Mr. Moore.—I have received a letter from her. I have not seen her.

Mr. Miller.—I beg the Commissioner to see the lady. She wishes the inquiry to be adjourned; and I am not in a situation to examine the witnesses.

Commissioner.—You shall have the witnesses again.

Mr. Miller.—She has been a resident at Guernsey all her life.

Commissioner.—Mr. Temple, do you propose to carry it back to any period of her residence in Guernsey?

Mr. Temple.—I do not. I only wish to carry it back to the day of her own documents in England. She has not been latterly domiciled at Guernsey. She has not lived there for some years. She has, as I understand, lived at Rome, and other places.

Commissioner.—I have received a letter from the alleged lunatics.

Mr. Miller.—And I should like that letter to be read to the Jury.

The Commissioner.—Certainly. I have no objection to do so. "Maria and Amelia Collings protest against the haste with which the Commission has been got up, for the purpose of transferring their property into the hands of Mr. and Mrs. Findlater, and solicit they may have a month's adjournment ere judgment be pronounced."

Mr. Miller.—Well, I think nothing can be more rational.

Commissioner.—Go and see her, if you like, and if you, as her counsel, tell us on your return that you are of opinion an adjournment is absolutely necessary, it will then be for me to consider your application.

Mr. Miller.—I must say the Commission appears to have been got up in much haste, and I beg to remind you of the case in Vesey, in which the Commission was quashed because an adjournment was refused.

Commissioner.—I am perfectly aware of that case. If she comes here to-day, and conducts herself rationally, and the jury consider her of sound mind, she ought to be set at liberty; but it will be for the jury to be guided as to her conduct by her answers and statement when she appears before them. We had better go on with the case, as I do not think this a proper time to ask for an adjournment.

A Juryman.—That letter is hardly the act of a lunatic.

Commissioner.—You had better not form any opinion on the letter, as it is not yet in evidence.

Mr. Miller.—I repeat that she presses very much for an adjournment, and she is anxious that the Commissioner should go down and see her.

(Mr. Miller with Mr. Moore here left the room and proceeded to the asylum, for the purpose of seeing Miss Maria Collings. On his return, he stated that the ladies would not leave each other, but were desirous, as he before stated, to have an adjournment.)

Commissioner.—We had better get on with the case in order to save expense, and we may afterwards adjourn if it becomes necessary.

Mr. Temple.—I appear on behalf of the petitioner, Mrs. Findlater, the only member of the family of the unfortunate lady, and I confess I did not think any difficulty would arise, as the lady was placed in confinement by the very parties who instruct my learned friend. Her father was a Captain Collings, of the 8th regiment of Native Madras Infantry in the East Indies, and he was married to the daughter of a Spanish merchant, Assando, by whom he had four children, Amelia, Maria, and Mrs. Findlater, and a son who died at the age of about five years. The daughters were sent over many years ago to their grandfather in this country, became natural subjects, and were entitled to the protection of the law of this country. Mr. and Mrs. Findlater, the sister and brother-in law of Miss Maria Collings, had been residing in Montpellier, in the south of France, and had only lately arrived in England, and when they arrived, they found them in a lunatic asylum. In the year 1847, Mr. Goodman, clerk to the Lord Mayor of London, received some letters, which will be put in evidence before you, and in which this lady states the most extraordinary case. She asserts that she is the heiress to the Spanish crown: that she is a descendant of Ferdinand the Seventh and the Princess Buorbon-de-Bourbon; that she is entitled to immense revenues, and that her mother has been assassinated in Guernsey, and that the people of that island are living upon her property; and praying that the Lord Mayor will apply to the Chancellor of the Exchequer on her behalf. If, after reading these letters, you have the slightest doubt of the state of her mind, for God's sake liberate her at once, as this is a proceeding not for the purpose of restraint, but for the object of her comfort and protection. The Lord Mayor kindly wrote to her relatives at Guernsey, and it was subsequently found necessary to place the unhappy lady in a private asylum. If you should be of opinion that commission was not necessary for her comfort and protection, nobody would rejoice more at such a result than Mr. and Mrs. Findlater, for whom I appear. I will now read you the letters written by Miss Collings in the order in which they were sent, as it will be a saving of time, and they may be better understood.

Mr. Miller.—Are you going to prove them?

Mr. Temple.—I shall read nothing to the jury that I am not going to put in evidence.

Commissioner.—I presume you are going to call Mr. Goodman to state when he received them. Is he here?

Mr. Temple.—I believe not; but I shall prove them to be in Miss Collings' handwriting. The first is dated the 4th of August, and is addressed to Mr. Goodman. It commences—

"The Princess Bourbon-de-Bourbon was the lawful child and heiress of that prince of Spain, who was unfairly treated by his parents and others. He resided several years in Hindostan, where he married a French lady of royal birth. Their daughter, Euphemia, was born in November 1795, at Pondichery; the prince called himself 'Arsando,' a Spanish captain and merchant. He kept a ship of his own, in which he sailed about the Indian ocean, and thus returned to Spain. His child was brought up in India as an orphan, by an English lady, and married in 1812, Lieutenant Collings, 8th Madras infantry. He was ignorant of her rank or fortune, as she was then portionless. He died at Hydrabad, 1818. On the day of his burial, his house and establishment were sold for the government, and widow and orphans were turned out destitute, and subsequently in poverty. Thus the widow, unprovided for, accepted a second marriage with Captain Alexander Thompson: they did not agree, and were divorced in three months. Our mother was obliged to remain in India, and Captain Thompson brought over her daughters to England. He died in 1824. Our brother died in India, aged five years. Our guardian, William Collings, wrote anxiously for his brother's son to be sent to him, and received answer that he was dead, and our mother would 'ere long join her children in England.'

"Euphemia De Bourbon, about this time, obtained possession of her inheritance and rank, and received the testament of the prince, her father. Maternal love induced her to seek her daughters ere she assumed the princely state.

"On the fourth day of her arrival in Guernsey, she was barbarously assassinated by blows struck by the stabs of knives; and a demoniac, named Nicholas Pedvin, asserts he dashed her to pieces.

"The assassin plot was concerted and executed by a band of smugglers, on account of the smuggling interests, and fraudulent enrichment of the Guernsey families and others, though instigated by one who expected secretly to seize the crown of Spain through a concerted plan. Previous and expectant of the arrival of our mother, they all feared that this beautiful princess would form another marriage, and the royal heirship pass away to other children. They heeded not her protestation that she would devote herself to her *daughters*, but compelled her to sign a will, by which her property fell under the power of the jurat William Collings, and became divided amongst his family and others. It was at that trying moment that the cry was overheard, 'O God, my Saviour, have pity on me!' They then effected to take her to see 'her dear Maria,' and led her like a lamb to the slaughter, from the house of Mr. Collings, on the fourth day of her arrival.

"A continual mystery, and system of deception, was necessarily kept up around her children; they heard Sir William Collings declare, 'That he knew very great secrets, which he would take care should never be revealed,' and by degrees the light broke upon them. The bailiff, royal court, and colleagues, decided they should be stigmatized as deranged, if they at all deranged their concerns; it becoming an evident arrangement of the cautious plans and proceedings of the past twenty years, and the knowledge they have gained from our stolen letters has enabled them to anticipate our measures.

"*August*, 1847." "HEIRS DE BOURBON."

You will find, gentlemen, that Mr. Goodman, by direction of the Lord Mayor, communicated with Mr. Collings, of Guernsey, who came over, and having obtained this certificate, placed the unfortunate lady in a private asylum. (A number of other letters, full of delusions, and extremely incoherent, were put in and read. All the letters contained conclusive evidence of her melancholy state of mind.) After this, Mr. and Mrs. Findlater came to England, and having taken the opinion of several eminent medical gentlemen as to her state of mind, this commission of inquiry was asked for and obtained.

Mr. MILLER again asked for an adjournment. This is the first we have heard of these letters, and for aught we know to the contrary, she may turn out to be related to the parties she refers to.

COMMISSIONER.—I presume they will show she is not related. The letters may be very true for what I know, but they have not been proved yet.

MEDICAL EVIDENCE.

Dr. FORBES WINSLOW examined.—I am proprietor of Sussex House Asylum, Hammersmith. Have had considerable experience in the treatment of the insane for many years, having had two establishments under my care for a long period. On the 8th of December last, I attended at the asylum, by direction of Mr. Rymer and Mr. Findlater, for the purpose of examining Miss Maria Collings. I referred to her property, when she told me she had large possessions. I asked her who had left them, and she said her mother. I asked her how she knew it, and she said she understood that large property had been left to her. I then asked her who had possession of it, and she said it had been taken possession of by Sir William Collings. She could not tell me the amount of her property, but she led me to suppose it was something very great. I then referred to her rank and position in life, and she said her mother was related to the Bourbon family. Upon being asked how she knew it, she said that it was her impression and belief, and that it was generally known to all parties in the Island of Guernsey; and that Lord Palmerston, and General Napier, the governor of the island, had proofs of her connexion with the royal family of Spain, and of her rights. On being asked if she had communicated this fact to any parties, she said she had written repeatedly to the Lord Chancellor on the subject; and referring to her mother, she represented that she had been cruelly murdered in the Island of Guernsey, in 1828. She said that her mother was taken to the retired part of the island, and there assassinated. On being asked how she became acquainted with the fact, she said it was generally believed by the whole Island of Guernsey; and on being asked who the parties were that were implicated in the murder, she said Sir William and Joseph Collings. That they instigated the murder, with the view of getting possession of her mother's property; and that previously to her mother's assassination, Sir William and Joseph Collings made her transfer all her property to them. From representations made to me as to her rank, I should consider she was labouring under delusions, and unfit to take care of herself. She was not violent. Assuming that she is not related to the family of Spain, and that she does not possess the property she represents herself to have, I consider her of unsound mind.

Cross-examined by Mr. MILLER.

Dr. WINSLOW.—It is common for persons having delusions of a certain character to manage their persons and property. I did not enter into an examination of Miss Collings' views upon general points. I am not aware that the lady entertains any particular notions upon religious matters. Her answers were connected; and there was not anything in her manner peculiar, but the apparent truthfulness and earnestness with which she made her statements, and which left an impression on my mind that she believed they were true.

Mr. MILLER.—If she were related to a grandee of Spain, and that led to the impression that she was connected with the royal family of Spain, would you consider that to be a delusion?

Dr. WINSLOW.—She might have been wrongly informed; and if she believed it, and had good evidence for the belief, that would be no delusion. In order that you may understand me, I will put a case. If A believes that she is entitled to a reasonable amount of property, to the extent of 10,000*l.* or 20,000*l.*, that might arise from misinformation, and I should not consider it a delusion; but if A believes that she is entitled to a very large amount, such as 100,000*l*. or 200,000*l*., or any other large sum, beyond the range of reason and probability, I should (in the absence of good evidence) entertain grave doubts as to the sanity of the person's mind.

Mr. Miller.—I understood you to say that she did not state the amount of her property?—No, she did not.

In reply to other questions, Dr. Winslow said that insanity and unsoundness of mind often consisted in the exaggeration of a fact; and it was no logical inference of the mind being in a sound state, because there existed some degree of evidence for what is pronounced to be an insane belief.

Mr. Miller.—You say this lady fancies herself to be related to the royal family of Spain. If she were related, would you still be of the same opinion?

Dr. Winslow.—Under these circumstances it would be a fact, not a delusion.

Mr. Miller.—Apart from these delusions, you consider her competent to take care of herself?

Dr. Winslow.—I have no evidence to the contrary.

Mr. Miller.—Persons with delusions manage themselves and property?

Dr. Winslow.—I have no doubt they do.

Re-examined by Mr. Temple.—I consider a delusion to be an unsound deduction from inadequate premises. I should say, the letters I have heard read are most conclusive evidence of very extensive unsoundness of mind. I consider her belief, as to the assassination of her mother, to be a very strong delusion.

Mr. Bowling examined.—I am a surgeon at Hammersmith, and have had twenty-five years' experience in cases of insanity. I accompanied Dr. Winslow to the asylum, on the 8th December, and saw Miss Collings there.

Mr. Temple.—I suppose it will not be necessary to go through all this again?

Mr. Miller.—Oh, certainly not. You have heard Dr. Winslow's statement; do you concur in his evidence?—Yes, I have no doubt she is of unsound mind.

Commissioner.—And unable to manage herself and her property?—Oh, yes, of course.

Cross-examined by Mr. Miller.—I have heard that the lady's property is 5000*l*., but she says she has got more than 100,000*l*. I think Dr. Winslow put the question to her. My belief of the unsoundness of her mind is formed from the lady having overstated her property. If she is entitled to very large property, then of course there is no delusion. I formed my opinion from the improbable nature of what she stated as facts.

Dr. Thomas Hodgkin, of Brooke-street, Grosvenor-square, examined by Mr. Temple.—I saw her a few days before she came into the asylum, the latter end of August. I have seen her repeatedly. She says her affairs are well known to Lord Palmerston, Lord John Russell, and other high personages, and that her connexion with the House of Spain is a matter of notoriety. I am not aware whether Maria gave herself any name. I believe her to be a person of unsound mind, and incapable of taking care of herself and her property. I have no doubt, not the smallest, that the letters are genuine. I have attended her in the house, as I have my other patients, three or four in number. I am not the regular attendant at the asylum. The letters were admitted by one of the sisters to be genuine. Those letters show delusions. The case is a very remarkable one. I never before knew two lunatics to agree in the same delusions. Maria says she is entitled to the whole of Guernsey. I do not think any harm could arise from the jury seeing her.

Mr. Mark Dewsnap, surgeon, examined by Mr. Temple.—I am regular medical attendant at the asylum. Miss Maria Collings came to the asylum on the 4th September last. I first saw her on the following day, and the last time on Saturday last. Her delusions are as to conspiracies to defraud her of her property, which she considers to be of large amount. She stated that her mother was the Princess of Spain, and went on to say that Louis Philippe had used her money to portion the Duke of Montpensier. She told me her sister was in communication with Lord John Russell, Lord Palmerston, and the Lord Mayor, all of whom she said were perfectly acquainted with her case. She said she considered herself under the protection of the premier, Lord John Russell, and that connected with her affairs at Guernsey, were Sir William Collings, and Mr. Joseph Collings.

By a Juror.—They are perfectly rational on all other subjects except their delusions.

Mr. Temple.—Dr. Winslow is desirous of correcting his evidence.

Dr. Winslow.—I find, upon reference to my notes, that she said 100,000*l*. were due to her from the traders of Guernsey, as well as to her sister.

Mr. MILLER.—She did not say more than 100,000*l.* ?

Dr. WINSLOW.—No; she mentioned 100,000*l.* I made my note at the moment in the room.

EXAMINATION OF RELATIVES.

Mr. FINDLATER examined.—All letters produced are in the handwriting of Miss Maria Collings. I received them from Mr. Currie. I married the sister of Maria Collings. The family consists of my wife and her two sisters. My wife's father was Captain Elias Collings, of the Indian army. Her mother was the daughter of a captain of his own vessel; his name was Arsando. Her mother died before my marriage, I believe in the year 1826 (it was in 1828). After her first husband's death, she was married to Captain Thompson, and she died at Madras. I never heard any imputation against any person that she was murdered. She was not related to the royal family of Spain, or to the Bourbon family. It is, perhaps, about six years and a half ago since Miss Collings left Guernsey. She afterwards came to Montpellier, and subsequently went to Rome, Switzerland, and other places on the continent; and in the spring of 1847 they came to Montpellier. They only came on the last occasion to visit us, and after a short stay, they set off to go to London. My wife was in the constant habit of corresponding with them, and we first heard of the state of her mind in the beginning of September. When we came to England, we found them in an asylum. When we first saw them, they held these delusions, but asserted they had proofs. Miss Maria Collings had 50*l.* per year in the Bank of England, besides a pension of 60*l.* from the Madras Orphan Military Asylum. Her property is in the hands of trustees. It altogether amounted to 199*l.* 16*s.* 2*d.* per year, besides some household property, &c. The property left to the three sisters was equally divided, and I can tell you what my wife's property is, but I cannot say what Miss Collings' may be, as I do not know what she may have disposed of. The property of my wife consists of 50*l.* 5*s.* 3*d.* per year in the Bank of England; 83*l.* 2*s.* 6*d.* in the French funds; 56*l.* in the Russian funds; and 10*l.* 8*s.* 5*d.* in the Neapolitan. She also has one-third of a house St. James's-street, Guernsey, one-third of thirty-five United States Bank shares, besides some rents. I believe Amelia, after she left Montpellier, went to Guernsey, to collect some evidence against her uncles.

Cross-examined by Mr. MILLER.—The property they have is in the hands of trustees, who are resident at Guernsey.

I believe they entertain very peculiar notions on religious subjects ?—They are what is called Swedenborgians.

Are you a licensed preacher ?—No, I am not.

Now, Mr. Findlater, do you mean to say you have not preached in the open air ?—Yes, I have preached in the open air.

Both in London and Guernsey ?—Yes.

Are you not an Irvingite ?—I do not acknowledge the word.

Do you not profess the unknown tongues ?—What do you mean by the unknown tongues ?

Have you not several times impressed upon them the religious principles of Mr. Irving ?—Those principles were taught them by their uncles and aunts long before I knew them.

Will you state what those principles are ?—Yes; if you will listen to me for half an hour, I have no objection to preach to you. (A laugh.)

Mr. TEMPLE objected to this line of examination, and the learned commissioner decided that it should not be farther pursued.

[An extract of the registry of the East India House was here put in evidence, showing that Euphemia Thompson, the mother of Miss Maria Collings, died at Madras, and not in Guernsey, as insisted upon by Miss Collings, and that she had no property in Spain.]

I believe there has been some dispute about the property ?—Sir William Collings required a release from my wife, without accounting for the property they were entitled to.

And was there not some mystery about the mother's death ?—She wrote from Madras, stating that she wished to join her children; and it is strange she did not come, for she was a fond and affectionate mother. It is supposed that Sir W. Collings prevented her coming from India.

So that all these circumstances might give rise to these peculiar delusions ?—Yes.

What is your opinion as to the competency of this lady to manage herself and her affairs ?

Mr. Temple.—I object to that question. The witness is called to speak to certain facts, and not to give his opinion. It will be for the jury to form an opinion upon the medical testimony.

Mr. Miller.—I believe you are a medical man ?—No ; I am only studying medicine.

Re-examined by Mr. Temple.—I graduated at Cambridge, and was private tutor to Sir Charles Burrell's son, and the son of Mr. Commissioner Shepherd. Myself and wife have always been on the very best of terms with both these ladies. When they took their leave of us at Montpellier, the greatest friendship existed between us.

By the Commissioner.—If Miss Maria Collings believes she has large wealth, that is not founded on fact. I have no reason whatever for believing that her mother died in Guernsey, or that she did not die a natural death. I never made any claim on the part of my wife for any money due to her in Spain. I might have done so had I believed she was entitled to any Spanish property. I have no reason to believe that she was connected with a grandee of Spain, or that she was in any way connected with any prince or princess of Spain, or in any way related to the Bourbon family.

The Commissioner.—What has she said to you about the death of her mother ?—She told me her mother came over to Guernsey, and four days afterwards was murdered ; a cousin of hers heard her shrieks ; and I believe the substance of her words was, that her uncle had immense sums belonging to her.

Mr. Temple now put in an additional letter, dated the 2nd of September, and addressed to the premier, Lord John Russell, commended to the care of Mr. Goodman. The letter in question afforded ample proof of Miss Collings' state of mind, and was signed "*Maria Hortensia de Bourbon.*"

Mr. Temple.—I have another letter which has not been opened, and, as it is addressed to the Lord Chancellor, I do not like to take upon myself the responsibility of opening it.

Commissioner.—Oh, give me the letter, as it is for the Chancellor, and I will open it, and share the responsibility with you.

The letter was then put in evidence. It was signed, Heirs De Bourbon, and identified as being in the handwriting of Miss Maria Collings.

### EXAMINATION OF THE LUNATIC.

The learned commissioner, with the counsel and jury, now proceeded to the asylum, for the purpose of examining Miss Maria Collings.

Commissioner.—I am afraid you think us rude, Miss Collings, so many gentlemen coming to see you, but we have come by direction of the Lord Chancellor, to ask you a few questions respecting your property. If there is anybody present you object to, they shall leave the room.

Miss Collings shook her head, and replied in the negative.

Commissioner.—Pray may I ask, Miss Collings, what property you have ?—It is already taken care of by Sir William Collings and Mr. Joseph Collings.

What is the amount of your property ?—You must ask my trustees who have had the management of it.

Do you know from whom you derived your property ?—It came from my ancestors the Collingses, as far as I am informed.

Do you know what has become of your mother ?—I believe she died in Guernsey. So the living witnesses say ; but we are not responsible for their statements. We endeavoured to find out the truth of the reports, and were shut up on so doing.

I have a certificate that she died in India. Here it is (producing the register).—This document I believe is forged. Any one can get such a piece of paper as that. I believe Joseph Collings saw her in Guernsey. She left India in 1828, and was not heard of after, but I do not see that this has anything to do with the state of my mind, and that I should be shut up.

Do you know the amount of your property ?—I believe 5000*l*. was settled on each of us ; and when we asked for an account from our guardians we were shut up. I don't see why Mr. Findlater should exercise a guardianship over us, when I am the elder sister.

In writing your letters, why do you sign yourself Heirs de Bourbon ?—I wrote to the Lord Chancellor. I knew of no other way to justify ourselves. I have been told that we are related to the family of Bourbon. You can go to the Governor, Napier, of Guernsey, or to the premier, Lord John Russell, or to Lord Palmerston, and ask them. They will state to you what is right. But what is the use of a lunatic, in a lunatic asylum, stating her impressions? Every one, I believe, is at liberty to think; it is a right we all have; and supposing that was our impression, we have lost our liberty for it.

But perhaps you can tell us?—You will wait, sir, and see what events take place. You will hear all about it if you write to the Premier, or if you wish to satisfy yourself, you can go to the offices of Lord Palmerston and Lord John Russell. Of course, whatever I say is insanity.

Perhaps they would think me rude?—That is no affair of mine. I tell you where you can satisfy yourself, and why trouble me? There is nothing so extraordinary in my thinking myself connected with the princess of Spain. You can inquire for yourself; it is of no use minding what I say, it is merely the evidence of a lunatic, as it is alleged. I suppose I am at liberty to think I belong to the royal family of Spain?

Had you not an impression that there was a conspiracy against you in Guernsey?—I had an impression that it was very rude towards my sister, when she was inquiring about the death of my mother, and had obtained the contents of two documents, for the people of the island to behave as they did to her. They sent her a summons to appear at the Royal Court of Guernsey; and an appeal was made to the Governor, who sent her a guard to protect her for one day, and she afterwards left the island.

And do you believe then, Miss Collings, that you are related to the Bourbon family?—I believe liberty of thought is allowed in England. We had a right to inquire further into our affairs, I suppose? We did not go running about the world, spreading these reports, without satisfying—

Then you did sign yourself, "Heirs de Bourbon?"—Yes; a sum of money got settled on the Montpensier family, and we used that signature to obtain particulars.

Was your father related to a Spanish grandee?—My father was an exile in India. He was a navigator, but a person of rank, who had been sent out of his own country.

Do you wish us to adjourn this case? If we do so, do you think you could prove what you have stated?—I think there has been much haste; I believe a month is generally allowed.

The commissioner, counsel, and jury then adjourned to the Roebuck Tavern.

Commission adjourned to February 1st.

### Adjourned Proceedings.

Mr. Temple, on the part of the Commission, called Dr. Winslow, of Sussex House, Hammersmith.

Mr. Temple.—On the former occasion you stated your opinion of the state of this young lady's mind. Have you seen her recently?—I saw her on Friday last, the 28th of January, and she remained in the same state of mind in which she was when I saw her before. I was with her about three quarters of an hour, and had a very long conversation with her. In the course of the conversation, she spoke in reference to Mr. and Mrs. Findlater in angry terms; and, in answer to my questions, she said that they had placed her in confinement, and wished to keep her so. I tried to explain; and, upon my stating to her that they had nothing to do with her present place of abode, she replied they had, for Mr. and Mrs. Findlater had placed her in confinement. I endeavoured to impress upon her mind that they had nothing to do with her confinement; but she seemed to be positive about it. She did not say upon whose representation it was that she arrived at these conclusions. She spoke of the murder of her mother, the same as she had done before; of her connexion with the Bourbon family, and of her immense wealth. She imagined that she was worth more than 100,000*l.*, and she said she was connected with the royal family of Spain; her mother had been cruelly murdered at the instigation of Sir William Collings, in order to get her money; that she was under the protection of the prime minister, the lord mayor, and Parliament. I was with her nearly three quarters of an hour before she would admit any of these delusions; and she told me that I had no business to ask her questions. I told her that I came with authority to communicate with her, and it was necessary for her to state what her belief was: and then she admitted all these delusions. It was at least half an hour before she would admit anything at all. I stated that I came by the authority of the chancellor. She did not refer with any degree of bitterness to the issuing out of this commission against her.

Commissioner.—Did you see her between the last time we met here and the 28th?—I have seen her once, and that was the 28th of last month.

Mr. Temple.—Did you apply to see her at any other time?—I applied to see her before, but could not see her. An appointment was made, and I was to have met Mr.

Bowling, but he did not come. She referred to Mr. and Mrs. Findlater, and I stated to her that they had not placed her in confinement; when she said that she knew they had, because she had been informed so.

Mr. Miller.—Are you sure she used the words, "under the protection of Parliament?"—Quite.

Mr. Miller.—Did she say that she was "under the protection of the prime minister?"—Yes, she did. I am quite certain of her having used the words.

Commissioner.—Did she say that her property was under the protection of the premier?—No; she said that she herself was under the protection of the premier.

Mr. Temple.—Have you any doubt as to her state of mind?—I have not the remotest doubt as to her being of unsound mind. In speaking of some of her relatives, and in reference to Sir William Collings being connected with the murder of her mother, she said that Sir William Collings and others were cheats and impostors.

Mr. Temple.—Is it a common feeling for persons in an unsound state of mind, to have bitter feelings against their most dear and near friends?—It is; those that they formerly most tenderly loved they often hate the most when the mind becomes deranged.

Mr. Miller.—Particularly if they are the parties who have taken out a commission against them. (Laughter.)

Commissioner.—Is that a common feeling in lunacy—a feeling of dislike to those that they naturally love?—It is a very common feature. I see it manifested in patients almost every day.

Mr. John Bowling.—I saw Miss Maria Collings on Sunday last, the day before yesterday; and perhaps I ought to state that I saw the two sisters together; and by my evidence you will find that they chimed in together in everything that was said. Amelia was excessively loquacious; and she answered those questions that were put to Maria; and, therefore, I was obliged to apply to Maria to know if they were correct. I saw nothing but what would confirm my previous opinion. I think Maria was rather more excited than she was on the previous occasion—that I attributed to the presence of her sister.

Mr. Temple.—Did she say anything about the parties who had placed her at the asylum?—She did, and alluded to her relatives in bitter terms;—for instance, Amelia said that George Collings was a forger and a swindler.

Mr. Temple.—This was said in the presence of Maria, I suppose?—It was, and I asked Maria if it was quite true, and she said it was.

Mr. Miller.—Did you observe anything that would make you doubt what she stated before?

Mr. Bowling.—Nothing at all. What I saw only tended to confirm me in my opinion of her state of mind. I put this question to her (Maria). I first said to her—"You promised to write me a letter, but you have never done so." She said that she recollected that promise. I then told Maria of the 100,000*l.*, and Amelia said that she was entitled to more than a million. I turned to Maria, and asked her the question; and she stated that what her sister had said was quite correct.—There appears to be a strong attachment between the two sisters. Amelia is a person very easily excited. Maria is not so much so; she is more cautious and more capable of guard than the other one. Maria told me that more information upon the subject was to be obtained elsewhere. She stated, if the chancellor was not satisfied, he could apply to the premier and to Lord Palmerston, and they could give him the information. There were no delusions except on those points. She also said that her mother was the heiress of Charles IV.—the same delusion to which Dr. Winslow referred.

Mr. Temple then read several letters written by Maria Collings.

Dr. Hodgkin examined.

Mr. Temple.—When did you see Miss Maria Collings last, Dr. Hodgkin?—I saw her on Saturday last. I continue in the same opinion, that she remains under the same delusions, and still believe her, in fact, to be incapable of taking care of herself.

Mr. Temple.—Has the result of your observations been of such a character as to confirm your former opinion now, or to make you doubt it in any way?—I am quite confirmed in my opinion of her state of mind. I think her remaining in a state of insanity. I talked to her upon some subjects, and heard her say things that convinced me that she was in an unsound state of mind. The last time that I saw her, she considered herself no subject of the crown of England, but thought that she was a foreigner of some superior right. I spoke to her closely about her affairs, and she said it was nothing to do with her, she was no subject of the crown, but she was connected with some high family abroad. The sisters appeared to be very much attached to each other; in fact, so much so, that I thought it was advisable that they should remain together, although I had formerly considered this undesirable, with regard to their delusions.

Commissioner.—You say you think this lady is subject to delusions, and that she is not in her right mind. Now, do you think that the effect produced upon the mind of Maria, may be from the *alleged* insanity of the other?—I have no doubt but that it is the case; and with a feeling to improve their state of mind, they mutually keep up these delusions: they disease each other.

Commissioner.—If Maria were separated from her sister, could she manage her affairs?—I cannot say. I should think her a very unlikely person to manage her own affairs.

The Jury.—Do you consider persons who are labouring under delusions capable of taking care of their own affairs?—There are some persons who take care of their own affairs while labouring under some delusions, but I cannot say that the delusions are so strong as they are in the present case.

Mr. Miller.—Now, suppose a person believed that any one under the influence of Mesmerism could read a book in the next room, what would you call that? Should you think it a delusion?—Yes, I should. I do not think this lady is a fit person to be left without control.

Mr. Miller addressed the Court at some length, after which the Commissioner went carefully through the evidence.

After twenty minutes' deliberation, the jury pronounced the following as the verdict:—

"We, the undersigned jurymen, are unanimously of opinion that Miss Maria Collings is of sound mind, and perfectly capable of taking care of herself and her property.

"Mr. Thomas Cock.

William Smith.

Thomas Parvell.

David Fitzgerald.

Henry Whitlock.

Samuel Mercer.

George Forster.

Mr. Samuel Spencer.

William Newcomb.

James Gale.

Charles Whittingham.

John Dyper.

Charles Edgmon."

Six gentlemen on the panel were dissentients to the verdict. The report of the Commission on Miss Amelia Collings (who labours under the same delusions) will appear in our next number.

---

